# A single point mutation converts a glutaryl-7-aminocephalosporanic acid acylase into an *N*-acyl-homoserine lactone acylase

**DOI:** 10.1007/s10529-021-03135-9

**Published:** 2021-04-23

**Authors:** Shereen A. Murugayah, Gary B. Evans, Joel D. A. Tyndall, Monica L. Gerth

**Affiliations:** 1 Department of Biochemistry, University of Otago, Dunedin, 9054 New Zealand; 2 The Ferrier Research Institute, Victoria University of Wellington, Petone, 5046 New Zealand; 3 School of Pharmacy, University of Otago, Dunedin, 9054 New Zealand; 4 Present Address: School of Biological Sciences, Victoria University of Wellington, PO Box 600, Wellington, 6140 New Zealand

**Keywords:** Glutaryl-7-aminocephalosporanic acid acylase, *N*-acyl-homoserine lactone acylase, Protein engineering, Quorum quenching, Site‐saturation mutagenesis

## Abstract

**Objective:**

To change the specificity of a glutaryl-7-aminocephalosporanic acid acylase (GCA) towards *N*-acyl homoserine lactones (AHLs; quorum sensing signalling molecules) by site-directed mutagenesis.

**Results:**

Seven residues were identified by analysis of existing crystal structures as potential determinants of substrate specificity. Site-saturation mutagenesis libraries were created for each of the seven selected positions. High-throughput activity screening of each library identified two variants—Arg255Ala, Arg255Gly—with new activities towards *N*-acyl homoserine lactone substrates. Structural modelling of the Arg255Gly mutation suggests that the smaller side-chain of glycine (as compared to arginine in the wild-type enzyme) avoids a key clash with the acyl group of the *N*-acyl homoserine lactone substrate.

**Conclusions:**

Mutation of a single amino acid residue successfully converted a GCA (with no detectable activity against AHLs) into an AHL acylase. This approach may be useful for further engineering of ‘quorum quenching’ enzymes.

**Supplementary Information:**

The online version contains supplementary material available at 10.1007/s10529-021-03135-9.

## Introduction

Many pathogenic bacteria use quorum sensing to regulate processes associated with virulence and biofilm formation. Methods for disrupting quorum sensing (referred to as ‘quorum quenching’) are being widely explored as an alternative to traditional antibiotics for the control of infections and/or biofilm formation (Grandclément et al. 2015). In particular, there is a growing interest in the use of enzymes that can degrade quorum-sensing signalling molecules (Fetzner 2015).

One major class of quorum quenching enzymes is *N*-acyl homoserine lactone (AHL) acylases. These enzymes irreversibly degrade AHLs, which are key quorum sensing signals produced by Gram-negative bacteria (including many pathogens). AHLs can vary in the length of the acyl side chain (usually 4–18 carbons) and substituents (e.g. 3-oxo or hydroxyl group).

Naturally occurring AHL acylases have shown promising quorum quenching abilities both in vitro and in vivo (Bokhove et al. 2010; Liu et al. 2017; Sio et al. 2006; Wahjudi et al. 2011). However, there remains a great deal of interest in engineering quorum quenching enzymes to alter their specificity and/or stability for potential applications in human health, biotechnology and/or agriculture (Billot et al. 2020; Murugayah and Gerth 2019).

Most AHL acylases are part of the N-terminal nucleophile hydrolase family (Bokhove et al. 2010; Utari et al. 2017). This family of enzymes also includes glutaryl-7-aminocephalosporanic acid acylases (GCAs). GL7-ACA acylases (GCAs) are important enzymes for the production of semisynthetic cephalosporin antibiotics. Natively, these enzymes convert glutaryl-7-aminocephalosporanic acid (GL7-ACA) into 7-aminocephalosporanic acid (7-ACA).

In this study, we have used the GCA of *Pseudomonas* sp. strain SY-77 (Kim et al. 2000) as a scaffold for engineering an enzyme with AHL acylase activity. This GCA was chosen as it has been shown to be amenable to engineering (Isogai and Nakayama 2016; Otten et al. 2002; Sio et al. 2002) immobilisation (Lee et al. 2002) and production under industrial fermentation conditions (Conti et al. 2014). It has the same structural fold (αββα sandwich fold) and uses the same N-terminal nucleophilic amino acid (Ser) as AHL acylases. Despite these similarities, it has no native activity towards AHLs (Gasteiger et al. 2005; Murugayah et al. 2019; Sio et al. 2006).

We used a combination of site-saturation mutagenesis and high-throughput screening to identify a variant (Arg255Gly) with new activity towards long-chain AHLs and complete loss of activity towards its native substrate (GL7-ACA). Our study provides insights into the amino acid residues that dictate the specificity of GCAs and highlights the potential of these enzymes as scaffolds for engineering.

## Materials and methods

Additional details are provided in the Supplementary Information.

### Library construction

A previously constructed plasmid for the expression of the *Pseudomonas* sp. strain SY-77 GL7-ACA acylase (GCA) gene was used as the starting template for mutagenesis (Murugayah et al. 2019). This plasmid (pET20-GCA-His6) encodes a truncated form of the GCA gene (without the signal sequence) and a C-terminal His_6_ tag. Seven site-saturation mutagenesis libraries were constructed using overlap extension PCR with NNK or 22c degenerate primers (Supplementary Methods and Supplementary Table 1). Conventional saturation mutagenesis using NNK primers was used for the first four libraries constructed (i.e. R255X, L222X, Q248X and F375X). The remaining libraries (i.e. Met174X, Tyr178X and Met347X) were constructed using a newer technique—the 22c method—which reduces codon redundancy and therefore also reduces the downstream screening effort (Kille et al. 2013). The resulting PCR products were subcloned back into pET20 and the plasmids used to transform the expression strain *E. coli* BL21-Gold(DE3) by electroporation.

For each library, 91 colonies were picked into a 96-well microplate with 100 µl LB ampicillin (100 µg ml^−1^) per well. In addition to the library variants, the following controls were included the remaining wells of each plate: pET20-GCA-His_6_, *E. coli* BL21-Gold(DE3); *E. coli* BL21 (DE3); pD441-Pa0305 *E. coli* C41(DE3) [AHL acylase positive control (Murugayah et al. 2019; Wahjudi et al. 2011)]; *E. coli* C41(DE3). After 18 h incubation at 28 °C, glycerol was added to each well to a final concentration of 15 % (v/v) and the plates were stored at − 80 °C. Library diversity was confirmed by sequencing 10 randomly selected transformants from each library.

### Library expression and purification

High-throughput protein expression was done in deep-well microplates containing 1 ml autoinduction media. After expression, the cells were lysed, and the proteins purified using HisPur Cobalt Spin Plates (Thermo Fisher Scientific) according to manufacturer instructions except that the elution buffer contained 75 mM imidazole instead of the recommended 150 mM imidazole [note: higher concentrations of imidazole can interfere with the downstream fluorescamine-based activity assay (Murugayah et al. 2019)]. Three independent purifications were performed for each library.

### High‐throughput library activity screening

The purified proteins from each library were assayed as described previously (Murugayah et al. 2019). Assays were conducted in 96-well flat-bottom black microplates containing 10 µl purified protein and 190 µl of either GL7-ACA or pooled substrate pairs (e.g. C4- and C6-HSL, C8- and C10-HSL, or C12- and 3-oxo-C12-HSL). The plates were incubated at 30 °C with shaking at 100 rpm for 24 h then fluorescamine was added to a final concentration of 1 mM. The relative fluorescence in each well was measured using a CLARIOstar Microplate Reader (BMG LabTech) with the gain set using 200 µM of the reaction product (i.e. 7-ACA or HSL).

### Large‐scale acylase expression and purification

Cells were grown in 500 ml AIM-TB at 37 °C with 200 rpm shaking. At OD_600_ 0.7, the cultures were shifted to 18 °C and incubated for an additional 30 h with 200 rpm shaking. Cells were harvested by centrifugation (3000×*g*, 15 min, 4 °C) then resuspended in lysis buffer. Cells were lysed by sonication and the proteins were purified using TALON Metal Affinity Resin (Clontech) according to the manufacturer instructions. After purification, the proteins were exchanged into storage buffer (50 mM potassium phosphate, 200 mM sodium chloride, 10% w/v glycerol pH 7) using 10 kDa molecular weight cut-off Amicon Ultra centrifugal filter units (Merck). Protein concentrations were measured using absorbance at 280 nm [ε_280_ = 135,680 M^−1^ cm^−1^ for wild-type GCA and the Arg255Gly variant, calculated using ProtParam (Gasteiger et al. 2005)].

### Determination of specific enzyme activity

The specific activities of the purified variants were conducted as described above, with 100 µM AHL and 100 nM purified protein. To calculate specific enzyme activity, the relative fluorescence units obtained were converted to amounts of product in µmol using an HSL standard curve. These values were divided by the assay period in minutes and the amount of protein in mg to calculate the specific enzyme activity (µmol/min/mg).

### Computational modelling

Docking simulations were carried out using the Genetic Optimisation for Ligand Docking (GOLD) program (Jones et al. 1997). Water molecules were first removed from the GCA structure (PDB 1OR0) and the Arg255 residue was mutated to Gly using PyMol (Version 1.8, Schrödinger, LLC). The oxygen atom on the side-chain of Ser199 was used as the centre of the binding site with a radius of 10 Å. 3-oxo-C12-HSL (PubChem CID 127,864) was obtained from the PubChem Compound Database (Kim et al. 2015) for docking. This was docked into the Arg255Gly model ten times at the automatic docking speed, where GOLD calculates the optimal number of times for the operation of its algorithm. Solutions were scored using the default CHEMPLP function and the best solution was analysed.

### Biofilm growth and crystal violet assays

Biofilms of *P. aeruginosa* were grown in M63 minimal medium [supplemented with arginine to a final concentration of 0.04 % (w/v)] on the pegs of Calgary Biofilm Devices (Innovotech, Canada). First, overnight cultures of *P. aeruginosa* were grown in LB with 300 rpm shaking. Next, overnight cultures were diluted 1:100 in M63 supplemented with arginine to a final concentration of 0.04% (w/v). Filter-sterilised enzyme was added to each well to a final concentration of 100 nM. The same volume of sterile buffer was added to an untreated well as a control. Biofilms were grown for 24 h at 37 °C (without shaking). Fresh media and enzyme were added and biofilms were grown for a further 24 h. To quantify biofilm mass, the biofilms were stained with crystal violet and the pegs transferred to plates containing 180 µl of 33% (v/v) acetic acid then sonicated for 15 min to solubilise the stained biofilms. The solubilised biofilms were transferred to a transparent microplate and absorbance at 590 nm measured.

## Results

### Structure‐based mutagenesis of GL7-ACA acylase

**Fig. 1:**
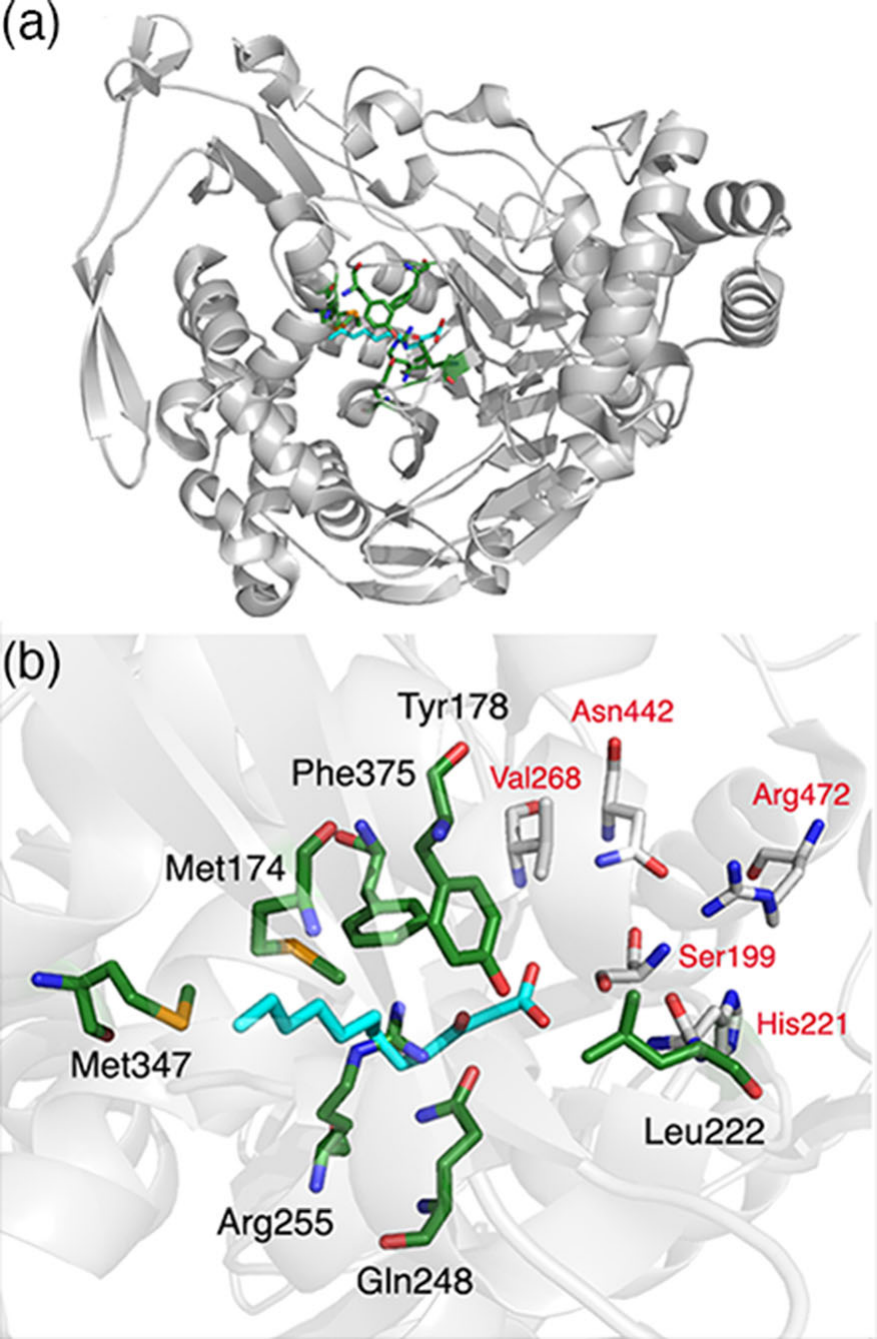
Selection of GCA residues for mutagenesis **a** Ribbon diagram of the overall structural fold of GCA (grey) with residues selected for mutagenesis shown as green sticks. 3-oxododecanoic acid (3-oxo-C12; cyan sticks) was modelled into the GCA structure by aligning it with the 3-oxo-C12 bound-PvdQ structure. **b** The GCA active site with 3-oxo-C12 (cyan sticks). Non-catalytic residues within a 5 Å radius of the modelled substrate were chosen for mutagenesis; these are shown as sticks with green backbones. Residues that are critical for catalysis are shown as sticks with white backbones. PDB entries for GCA [1OR0 (Kim et al. 2000)] and PvdQ [PDB 2WYC (Bokhove et al. 2010)] were used to construct the figure using PyMOL (Schrödinger, LLC)

To identify residues for randomisation, we aligned the structure of the *P. aeruginosa* PAO1 AHL acylase PvdQ (in complex with the product 3-oxo-C12) and the structure of GCA (Fig. 1 and Supplementary Fig. 1). The two structures align with a root mean square deviation of 2.2 Å. Based on this structural alignment, seven residues within a 5 Å radius of the aligned 3-oxo-C12 were chosen for randomisation: Met174, Tyr178, Leu222, Gln248, Arg255, Met347, Phe375 (Fig. 1b). Residues within the 5 Å radius of the substrate, but known to be critical for catalysis (i.e. Ser199, His221, Asn442 and Arg472), were excluded from mutagenesis (Fig. 1b).

Site-saturation mutagenesis was used to generate libraries consisting the seven selected positions. We picked and screened 91 randomly selected clones from each library, giving a $\ge$ 94 % chance that all 20 amino acids would be sampled at the mutated position, as calculated by GLUE-IT (Firth and Patrick 2008).

The selected variants from each library (637 variants in total) were purified and screened for activity towards six AHL substrates pooled in pairs (C4- and C6-, C8- and C10-, C12- and 3-oxo-C12-HSL) and the native substrate GL7-ACA, using a high-throughput fluorescamine-based assay (Murugayah et al. 2019). Of the seven libraries screened, AHL acylase activity was only observed in the Arg255X library. The lack of observed activity from the other six variant libraries could be due to a number of factors, including impaired catalytic efficiency, protein expression, solubility and/or processing. However, two variants from the Arg255X library – Arg255Ala, Arg255Gly—had activity in both the pooled C8- and C10-HSL screen and the pooled C12- and 3-oxo-C12-HSL screens (Supplementary Fig. 2).

### Characterisation of Arg255Ala and Arg255Gly variants

Next, the Arg255Ala and Arg255Gly variants were over-expressed and purified and their specific activities towards each substrate were determined. The specific enzyme activities are shown in Table 1. Neither variant had detectable activity towards the shorter-chain AHLs (C4, C6, C8-HSL) or the native substrate GL7-ACA acylase.

**Table 1:** Specific activities of Arg255Ala and Arg255Gly in µmol HSL min^−1^ mg^−1^

Substrate	Arg255Ala	Arg255Gly
GL7-ACA	n.d.	n.d.
C10-HSL	(1.1 ± 0.3) × 10^−5^	(1.8 ± 0.2) × 10^−5^
C12-HSL	n.d.	(2.6 ± 0.3) × 10^−5^
3-oxo-C12-HSL	n.d.	(9.4 ± 0.8) × 10^−7^

*n.d.* not detected

### Modelling of the Arg255Gly variant

**Fig. 2:**
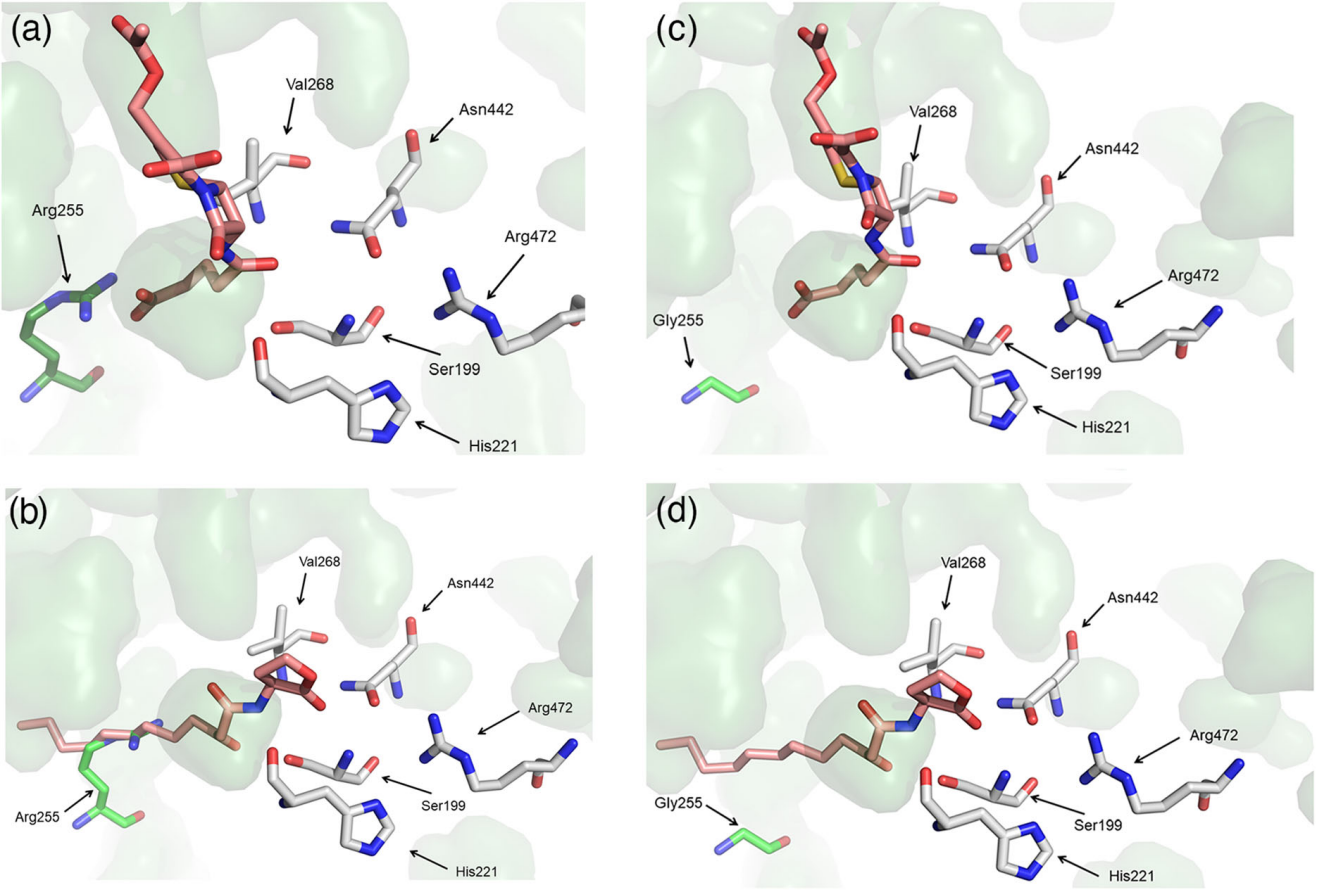
Modelling of the active sites of with GCA and the Arg255Gly variant. **a** Wild-type GCA with its native GL7-ACA substrate. **b** Wild-type GCA with 3-oxo-C12-HSL. **c** Arg255Gly GL7-ACA. **d** Arg255Gly with 3-oxo-C12. Residue 255 is shown as green sticks. Substrates (GL7-ACA and 3-oxo-C12-HSL) are shown as pink sticks. Residues that are critical for catalysis are shown as sticks with white backbones

To provide insights into the structural changes that led to the new activity towards AHLs, we attempted to crystallise the Arg255Gly variant. However, we were unable to obtain diffraction quality crystals. Therefore, we modelled the Arg255Gly variant with 3-oxo-C12-HSL to understand the structural basis of the new AHL acylase activity. As shown in Fig. 2a, the Arg255 side-chain of the wild-type GCA points into the active site, interacting with the glutaryl group of the native GL7-ACA substrate via electrostatic interaction. However, this Arg255 side-chain is predicted to clash with the 3-oxo-C12-HSL fatty acyl group (Fig. 2b). Mutation of Arg255 to glycine relieves this clash (Fig. 2d) but also abolishes activity toward the native substrate. This is most likely due to the loss of the electrostatic interaction with the removed sidechain (Gly) and the glutaryl group of the native GL7-ACA substrate (Fig. 2c).

### Enzyme treatment of *P. aeruginosa* biofilms

**Fig. 3:**
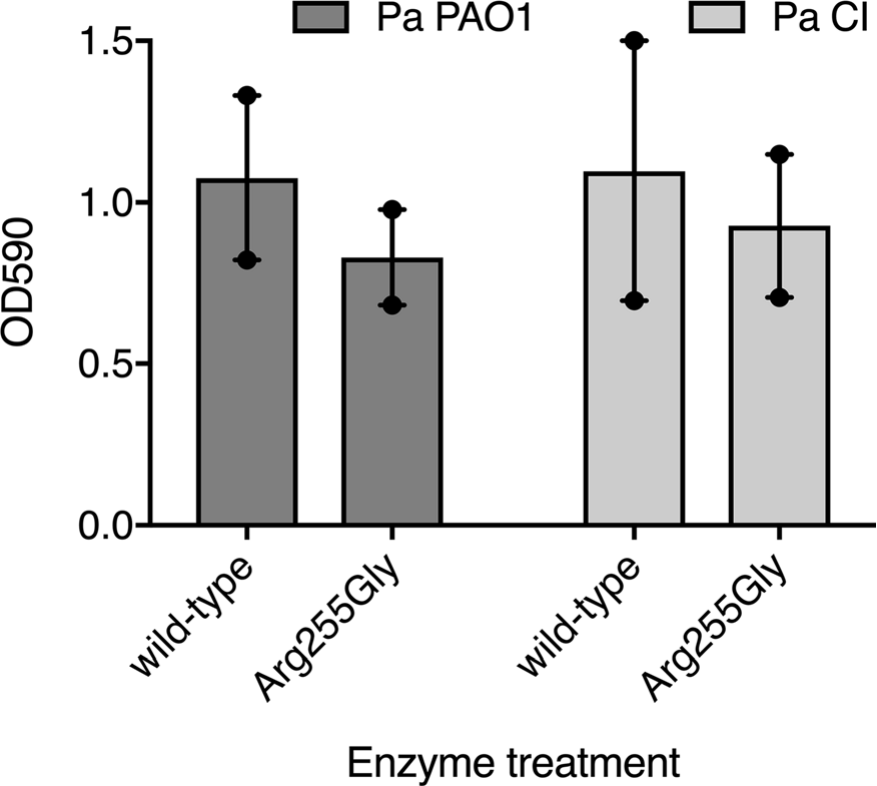
The effect of enzyme treatment on *P. aeruginosa* biofilm formation. Biofilms of *P. aeruginosa* PAO1 (Pa PAO1, dark grey) and *P. aeruginosa* clinical isolate (Pa CI, light grey) were grown in the presence of the wild-type GCA (wild-type) or Arg255Gly mutant (Arg255Gly) and biofilm formation was measured using crystal violet staining of the bacterial biomass. Two biological replicates were performed (n = 2) with four technical replicates each. The independent data points are shown as circles, bars represent the mean, and lines represent the range

We tested the Arg255Gly variant for quorum-quenching activity against biofilms of *P. aeruginosa*. We observed no considerable difference between biofilms treated with this variant compared to untreated biofilms (Fig. 3).

## Discussion

There is a great deal of interest in the engineering of quorum quenching enzymes due to their numerous potential biotechnological applications, including in human and animal health and/or agriculture (Billot et al. 2020; Murugayah and Gerth 2019). In our study, mutation of a single amino acid residue successfully converted a GCA (with no detectable activity against AHLs) into an AHL acylase. The Arg255Gly variant, which has weak *in vitro* activity against 3-oxo-C12-HSL *in vivo* (i.e. one of the major *P. aeruginosa* quorum-sensing signalling molecules), did not have sufficient quorum quenching activity to reduce biofilm formation of *P. aeruginosa*. However, the Arg255Gly variant had activity toward C10-HSL, which is a key signalling molecule for other microbial pathogens and also is strongly associated with biofouling (Billot et al. 2020; Tabraiz et al. 2020). Future work could explore the quorum quenching activity of this variant towards C10-HSL signalling. Alternatively, the Arg255Gly variant may be a useful template to engineer improved 3-oxo-C12-HSL acylase activity.

## Supplementary Information

Below is the link to the electronic supplementary material.

Supplementary file 1 (PDF 1226 kb)
