# Supplementary material for: A single point mutation converts a glutaryl-7-aminocephalosporanic acid acylase into an N-acyl-homoserine lactone acylase
Source: Biotechnol Lett. 2021 Apr 23;43(7):1467–73. doi: 10.1007/s10529-021-03135-9 (PMC8197700; doi:10.1007/s10529-021-03135-9)
Supplement: Supplementary file 1 — (PDF 1226 kb) [file 10529_2021_3135_MOESM1_ESM.pdf]

Supporting Information for:

A single point mutation converts a glutaryl-7-aminocephalosporanic acid acylase into an *N*-acyl-homoserine lactone acylase

Authors: Shereen A. Murugayah, Gary Evans, Joel Tyndall and Monica L. Gerth

Corresponding author: Monica Gerth (monica.gerth@vuw.ac.nz)

Supplementary Material and Methods

Materials

N-butanoyl-homoserine lactone (C4-HSL) and *N*-dodecanoyl-homoserine lactone (C12-HSL) were from Cayman Chemical. *N*-hexanoyl-homoserine lactone (C6-HSL), *N*-octanoyl-homoserine lactone (C8-HSL), *N*-decanoyl homoserine lactone (C10-HSL), *N*-3-oxo-dodecanoyl homoserine lactone (3-oxo-C12-HSL) and GL7-ACA were synthesised by Dr. Gary Evans (Ferrier Research Institute, Victoria University of Wellington). 7-amino cephalosporanic acid (7-ACA) and L-homoserine-lactone (HSL) were from Sigma-Aldrich. All stocks were prepared in 100 mM potassium phosphate buffer pH 7.0.

The clinical isolate of *Pseudomonas aeruginosa* was provided by Dr. Stephen Chambers (Department of Pathology and Biomedical Science, University of Otago). It was originally isolated from an infected knee implant and identified by 16sRNA sequencing and multilocus sequence typing.

Library construction

The degenerate primers used for library construction are shown in Supplementary Table 1. NNK site-saturation mutagenesis was used to construct the Leu222X, Gln248X, Arg255X and Phe375X libraries. For NNK site-saturation mutagenesis, each mix of forward and reverse primers encodes all 20 amino acids (where N = A/C/G/T, B = C/G/T, S = C/G, and K = G/T). For NNK mutagenesis, the theoretical completeness of a 91-variant library is 94% (Firth and Patrick 2008). For the remaining libraries – Met174Xm Try178X and Met347X, the 22c method was used to construct the libraries. This method requires more primers than NNK, but reduces codon redundancy and provides a more balanced distribution of codon per

amino acid (Kille et al. 2013). Primer design for 22c is described in Kille et al. Briefly, mixtures of six primers harbouring NDT, VHG, and TGG (forward) and AHN, CDB and CCA (reverse) were used per library. With this method, the theoretical completeness of a 91-variant library is 98%. For both libraries, 91 variants were randomly selected for high-throughput screening.

Library expression and purification

High-throughput protein expression was done in deep well microplates containing 1 mL autoinduction media (AIM-TB; AIM-Terrific Broth base including trace elements (Formedium) with 0.4% w/v glycerol and 100 µg/ml ampicillin) per well. The deep well microplates were incubated with 600 rpm shaking at 37 ˚C for 3 h, then at 18 °C for 30 h. The cells were harvested by centrifugation (3000 × g, 15 minutes, 4 °C) then lysed with 200 µL buffer (Kphos buffer: 50 mM potassium phosphate, 300 mM sodium chloride, 10% w/v glycerol, pH 7) supplemented with 0.5 mg/ml lysozyme, 2.5 U Benzonase Nuclease 250 U/µl (Merck), protease inhibitor cocktail (P8849; Sigma Aldrich) and 1× BugBuster Protein Extraction Reagent (Merck)). Insoluble fractions were pelleted (3000 × g, 15 min, 4 °C) and lysates were applied to HisPur Cobalt Spin Plates (Thermo Fisher Scientific) and purified according to manufacturer instructions. The proteins were eluted with Kphos buffer with 75 mM imidazole (higher concentrations of imidazole can interfere with the downstream fluorescamine-based activity assay (Murugayah et al. 2019)). Three independent purifications were performed for each library plate

Supplementary Table 1: Degenerate primers used for library construction.

Primer name	Sequence (5′ – 3′)	Purpose
R255X.for	CTCAGATCGGCCTGCCGGTGATT**NNK**TTTGCTTTCAATCAGCGT	NNK site-saturation mutagenesis of Arg255
R255X.rev	ACGCTGATTGAAAGCAAA**MNN**AATCACCGGCAGGCCGATCTGAG	
L222X.for	GTTGTTGCAGAATCCGCAC**NNK**AGCTGGACGACGGACTACT	NNK site-saturation mutagenesis of Leu222
L222X.rev	AGTAGTCCGTCGTCCAGCT**MNN**GTGCGGATTCTGCAACAAC	
Q248X.for	TGAAATCTACGGCGCTACT**NNK**ATCGGCCTGCCGGTGATTC	NNK site-saturation mutagenesis of Gln248
Q248X.rev	GAATCACCGGCAGGCCGAT**MNN**AGTAGCGCCGTAGATTTCA	
F375X.for	CCAGAATGCAAGTGCCGACC**NNK**AACATTGTGTACGCGGATCG	NNK site-saturation mutagenesis of Phe375
F375X.rev	CGATCCGCGTACACAATGTT**MNN**GGTCGGCACTTGCATTCTGG	
F375 22c NDT for	CGACC**NDT**AACATTGTGTACGCGGATCGTGAG	22c site-saturation mutagenesis of Phe375
F375 22c NDT rev	CAATGTT**AHN**GGTCGGCACTTGCATTCTGGCC	
F375 22c VHG for	CGACC**VHG**AACATTGTGTACGCGGATCGTGAG	
F375 22c VHG rev	CAATGTT**CDB**GGTCGGCACTTGCATTCTGGCC	
F375 22c TGG for	CGACC**TGG**AACATTGTGTACGCGGATCGTGAG	
F375 22c TGG rev	CAATGTT**CCA**GGTCGGCACTTGCATTCTGGCC	
M174 22c NDT for	GTCTG**NDT**AATTTTCTGTATGTCGCGTCCCC	22c site-saturation mutagenesis of Met174
M174 22c NDT rev	AGAAAATT**AHN**CAGACGGTGAGCGTGGGCA	
M174 22c VHG for	GTCTG**VHG**AATTTTCTGTATGTCGCGTCCCC	
M174 22c VHG rev	AGAAAATT**CDB**CAGACGGTGAGCGTGGGCA	
M174 22c TGG for	GTCTG**TGG**AATTTTCTGTATGTCGCGTCCCC	
M174 22c TGG rev	AGAAAATT**CCA**CAGACGGTGAGCGTGGGCA	
Y178 22c NDT for	TTCTG**NDT**GTCGCGTCCCCGGGTCGTACG	22c site-saturation mutagenesis of Tyr178
Y178 22c NDT rev	CGCGAC**AHN**CAGAAAATTCATCAGACGGTGAGCG	
Y178 22c VHG for	TTCTG**VHG**GTCGCGTCCCCGGGTCGTACG	
Y178 22c VHG rev	CGCGAC**CDB**CAGAAAATTCATCAGACGGTGAGCG	
Y178 22c TGG for	TTCTG**TGG**GTCGCGTCCCCGGGTCGTACG	
Y178 22c TGG rev	CGCGAC**CCA**CAGAAAATTCATCAGACGGTGAGC	
M347 22c NDT for	CGGGT**NDT**TTAGAGCAGTACTTCGATATGATCACCGC	22c site-saturation mutagenesis of Met347
M347 22c NDT rev	GCTCTAA**AHN**ACCCGGACGGTCCAGACCCG	
M347 22c VHG for	CGGGT**VHG**TTAGAGCAGTACTTCGATATGATCACCGC	
M347 22c VHG rev	GCTCTAA**CDB**ACCCGGACGGTCCAGACCCG	
M347 22c TGG for	CGGGT**TGG**TTAGAGCAGTACTTCGATATGATCACCGC	
M347 22c TGG rev	GCTCTAA**CCA**ACCCGGACGGTCCAGACCCG	

Supplementary Figure 1

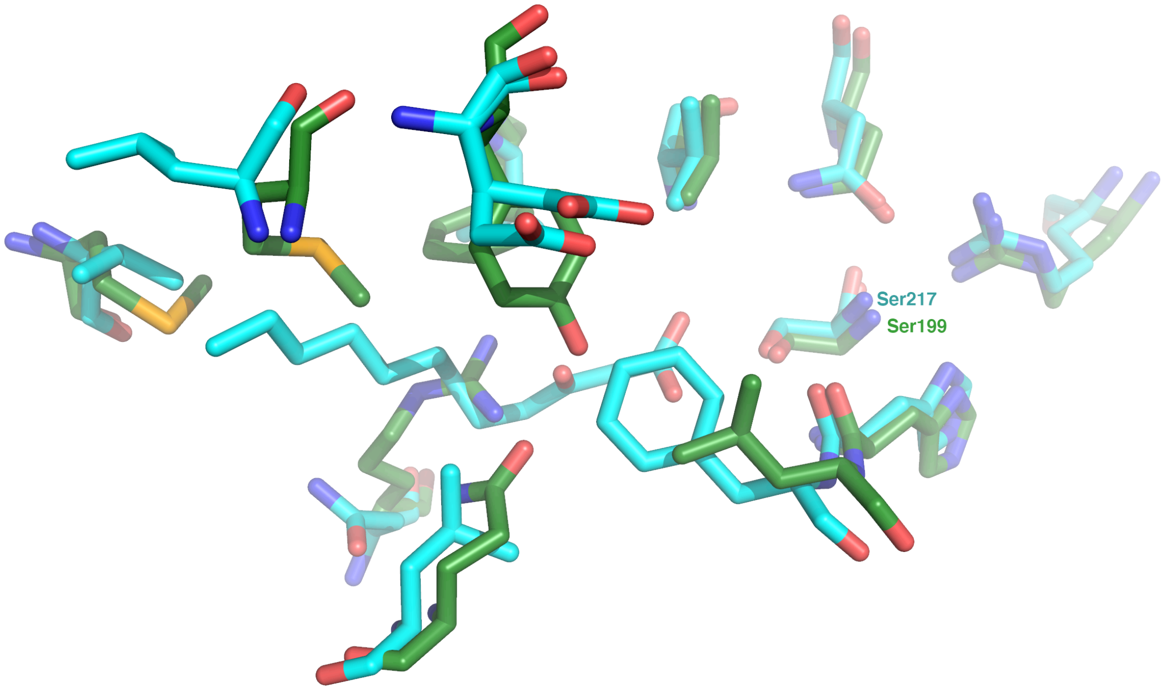

Supplementary Figure 1. Overlay of the GCA and PvdQ active sites. The GCA active site residues are shown as sticks with green backbones. PvdQ active site residues and 3-oxo-C12 are as shown as sticks with cyan backbones. Overall, residue numbers are omitted for clarity; however, the catalytic serine residue of both enzymes are noted for reference. PDB entries for GCA (1OR0 (Kim et al. 2000)) and PvdQ (PDB 2WYC (Bokhove et al. 2010)) were used to construct the figure using PyMOL (Schrödinger, LLC).

Supplementary Figure 2

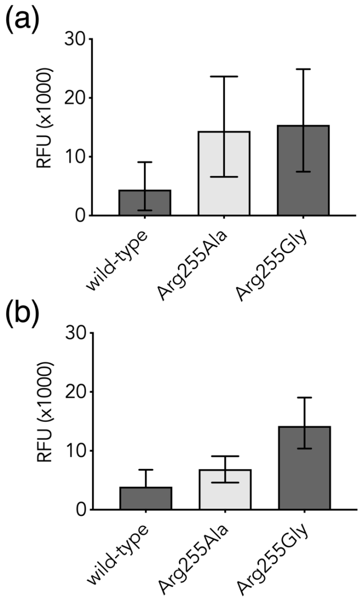

Figure S2. Screening results of lead variants from Arg255X site-saturation mutagenesis library against pooled substrate pairs. (a) C8- and C10-HSL (b) C12- and 3-oxo-C12-HSL. Bars represent the median, lines represent the range. n is the number of times variant occurred in each library: wildtype, n=5; Arg255Ala, n=7; Arg255Gly, n=5. RFU is the relative fluorescence units.

References

Bokhove M, Jimenez PN, Quax WJ, Dijkstra BW (2010) The quorum-quenching *N*-acyl homoserine lactone acylase PvdQ is an Ntn-hydrolase with an unusual substrate-binding pocket. Proc Natl Acad Sci USA 107:686-691. https://doi.org/10.1073/pnas.0911839107

Firth AE, Patrick WM (2008) GLUE-IT and PEDEL-AA: new programmes for analyzing protein diversity in randomized libraries. Nucleic Acids Res 36:W281-285. https://doi.org/10.1093/nar/gkn226

Kille S, Acevedo-Rocha CG, Parra LP, Zhang ZG, Opperman DJ, Reetz MT, Acevedo JP (2013) Reducing codon redundancy and screening effort of combinatorial protein libraries created by saturation mutagenesis. ACS Synth Biol 2:83-92. https://doi.org/10.1021/sb300037w

Kim Y, Yoon K-H, Khang Y, Turley S, Hol WG (2000) The 2.0 Å crystal structure of cephalosporin acylase. Structure 8:1059-1068. https://doi.org/10.1016/S0969-2126(00)00505-0

Murugayah SA, Warring SL, Gerth ML (2019) Optimisation of a high-throughput fluorescamine assay for detection of N-acyl-l-homoserine lactone acylase activity. Anal Biochem 566:10-12. https://doi.org/10.1016/j.ab.2018.10.029
